# Supplementary material for: Phylogenetic Distribution of WhiB- and Lsr2-Type Regulators in Actinobacteriophage Genomes
Source: Microbiol Spectr. 2021 Nov 24;9(3):e00727-21. doi: 10.1128/Spectrum.00727-21 (PMC8612146; doi:10.1128/Spectrum.00727-21)
Supplement: SUPPLEMENTAL FILE 1 — Supplemental material. Download SPECTRUM00727-21_Supp_1_seq4.pdf, PDF file, 1.4 MB [file spectrum00727-21_supp_1_seq4.pdf]

Supplementary Material

Title: Phylogenetic distribution of WhiB- and Lsr2-type regulators in actinobacteriophage genomes

Vikas Sharma[1*], Aël Hardy[1], Tom Luthe[1], and Julia Frunzke[1]*

[1]Institute of Bio- und Geosciences, IBG-1: Biotechnology, Forschungszentrum Jülich, 52425 Jülich, Germany

*Corresponding authors:

Vikas Sharma; Email: v.sharma@fz-juelich.de; Phone: +49 2461 612544

Julia Frunzke; Email: j.frunzke@fz-juelich.de; Phone: +49 2461 615430

Content:

Fig. S1: Correlation heatmap matrix of the thirteen identified regulatory protein domains within actinobacteriophages.

Fig. S2: Pairwise distance between different actinobacteriophage clusters calculated using Jensen-Shannon divergence method.

Fig.S3: Bar-plot displaying the distribution of actinobacteriophage genomes that encode two copies of *whiB* genes according to their host genus.

Fig. S4: Bar-plot representing the actual count and proportion of WhiB-encoding phages according to the known clusters.

Fig.S5: Dot-plot represents the global protein pairwise sequence identity between phage-encoded *whiB* gene copies per genome according to their host genus.

Fig. S6: Bar-plot displays the distribution of actinobacteriophage genomes that encode two copies of *lsr2* genes according to their host genus.

Fig. S7: Bar-plot representing the actual count and proportion of Lsr2--encoding phages according to the known clusters.

Fig.S8: Dot-plot represents the global protein pairwise sequence identity between phage-encoded *lsr2* gene copies per genome according to their host genus.

Fig. S9. Gene synteny plot showing the 5 kb regions flanking *lsr2.*

Fig. S10. Lsr2 homologs encoded by *Streptomyces* phages of the BE cluster are located in GC-rich, long direct terminal repeats

Fig. S11: WhiB phylogenetic subclade suggesting the acquisition of *whiB* by virulent *Streptomyces* phages from their host species.

Fig. S12: WhiB phylogeny subclade suggesting the acquisition of *whiB* by temperate *Mycobacterium* phages from their host species.

Fig. S13: Lsr2 phylogeny subclade suggesting the acquisition of *lsr2* by temperate *Streptomyces* phages from their hosts.

Fig. S14: Lsr2 phylogeny subclade suggesting the acquisition of *lsr2* by temperate *Gordonia* phages from their host species.

Fig.15: Lsr2 phylogeny subclade suggesting a transfer of *lsr2* from temperate *Gordonia* phages to *Mycobacterium* phages.

Legends of supplementary tables

Supplementary Figures

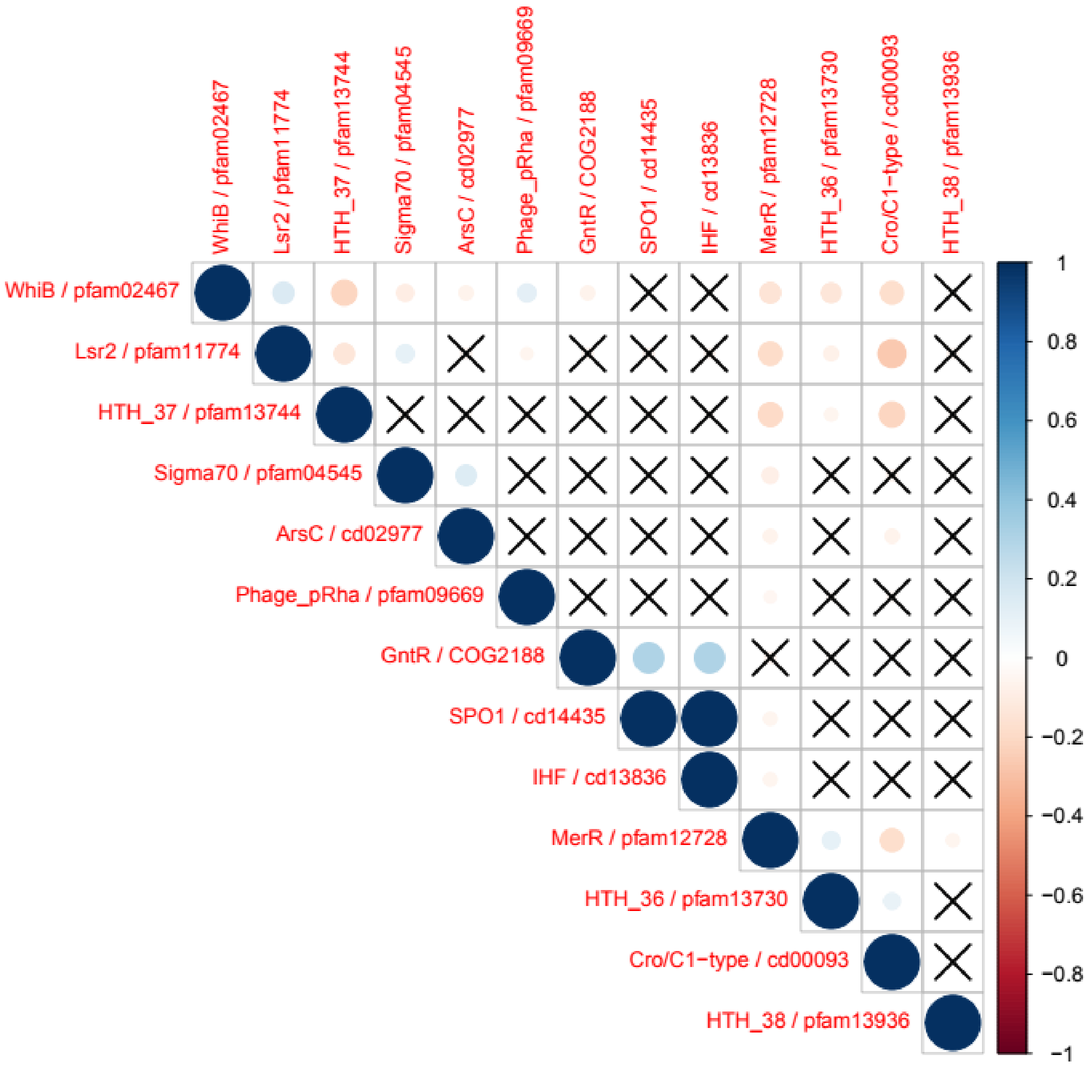

Fig. S1: Correlation heatmap matrix of the thirteen identified regulatory protein domains within actinobacteriophages. Circle color (Dark red: high negative correlation, Dark blue: high positive correlation) and the size indicate the correlation significance. Insignificant coefficients between the variables are denoted with the cross marks "X." Interestingly, this analysis revealed a negative correlation between Cro/CI and Lsr2 domains, suggesting that phage genomes rarely share these two different type of repressors.

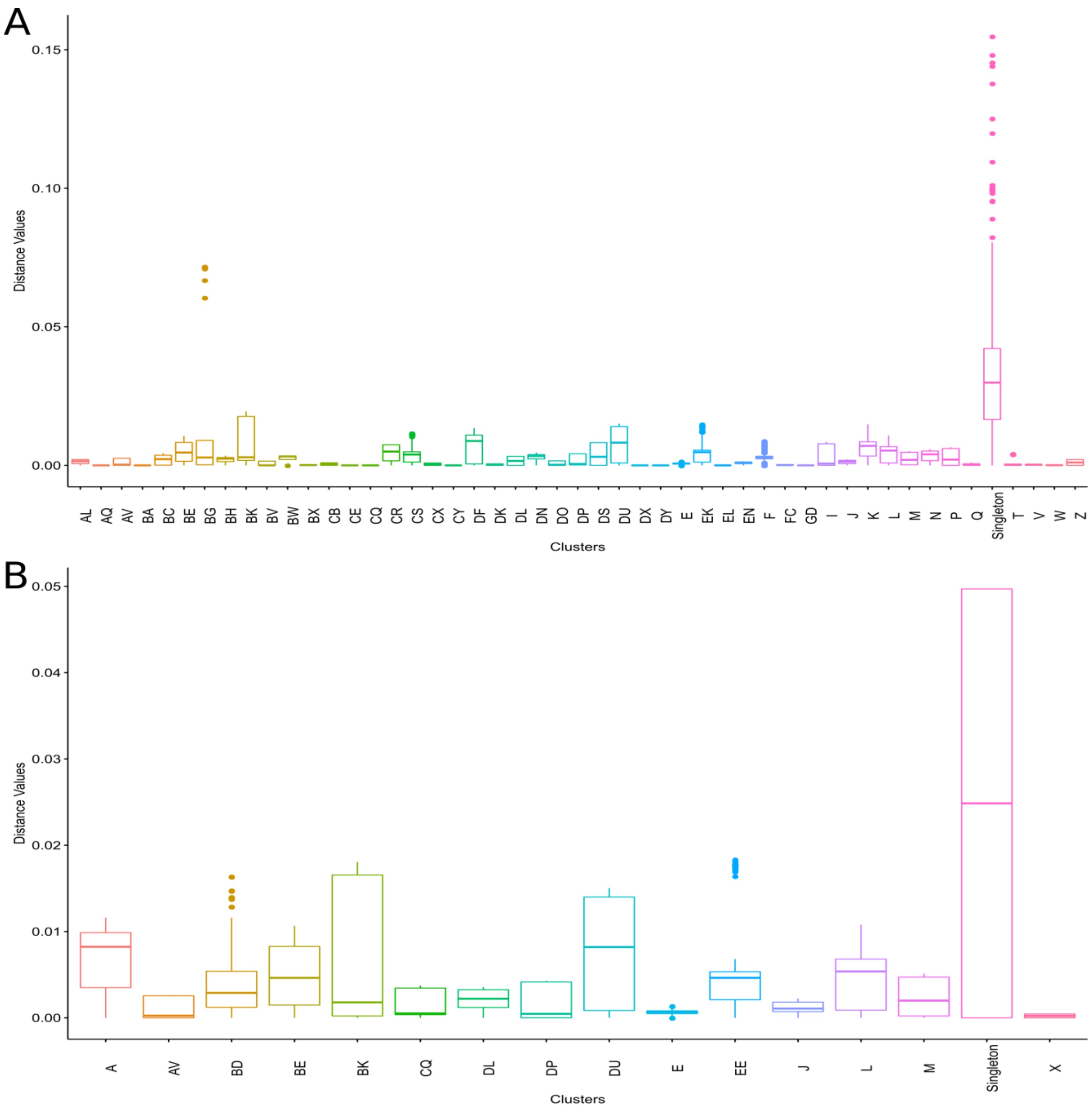

Fig. S2: Pairwise distance between different actinobacteriophage clusters calculated using Jensen-Shannon divergence method (1). A. Boxplot representation shows the pairwise distance between WhiB-encoding phages. B. Boxplot representation shows the pairwise distance between Lsr2-encoding phages.

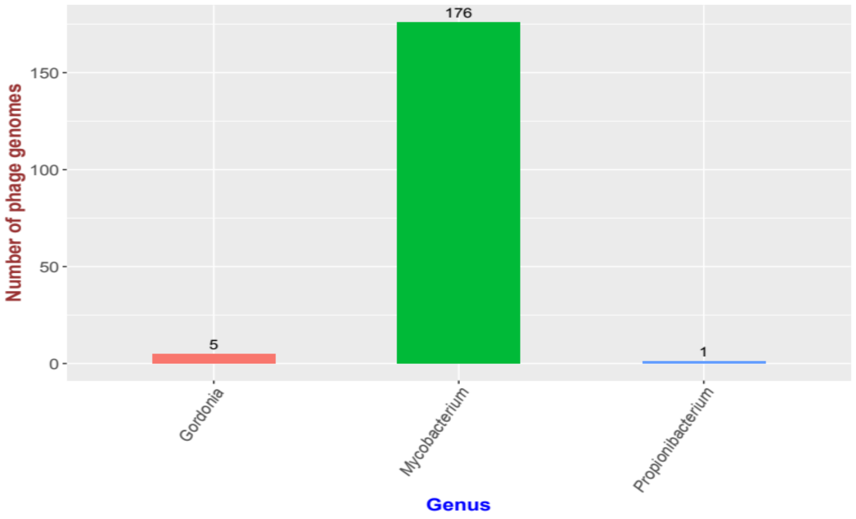

Fig.S3: Bar-plot displaying the distribution of actinobacteriophage genomes that encode two copies of *whiB* genes according to their host genus.

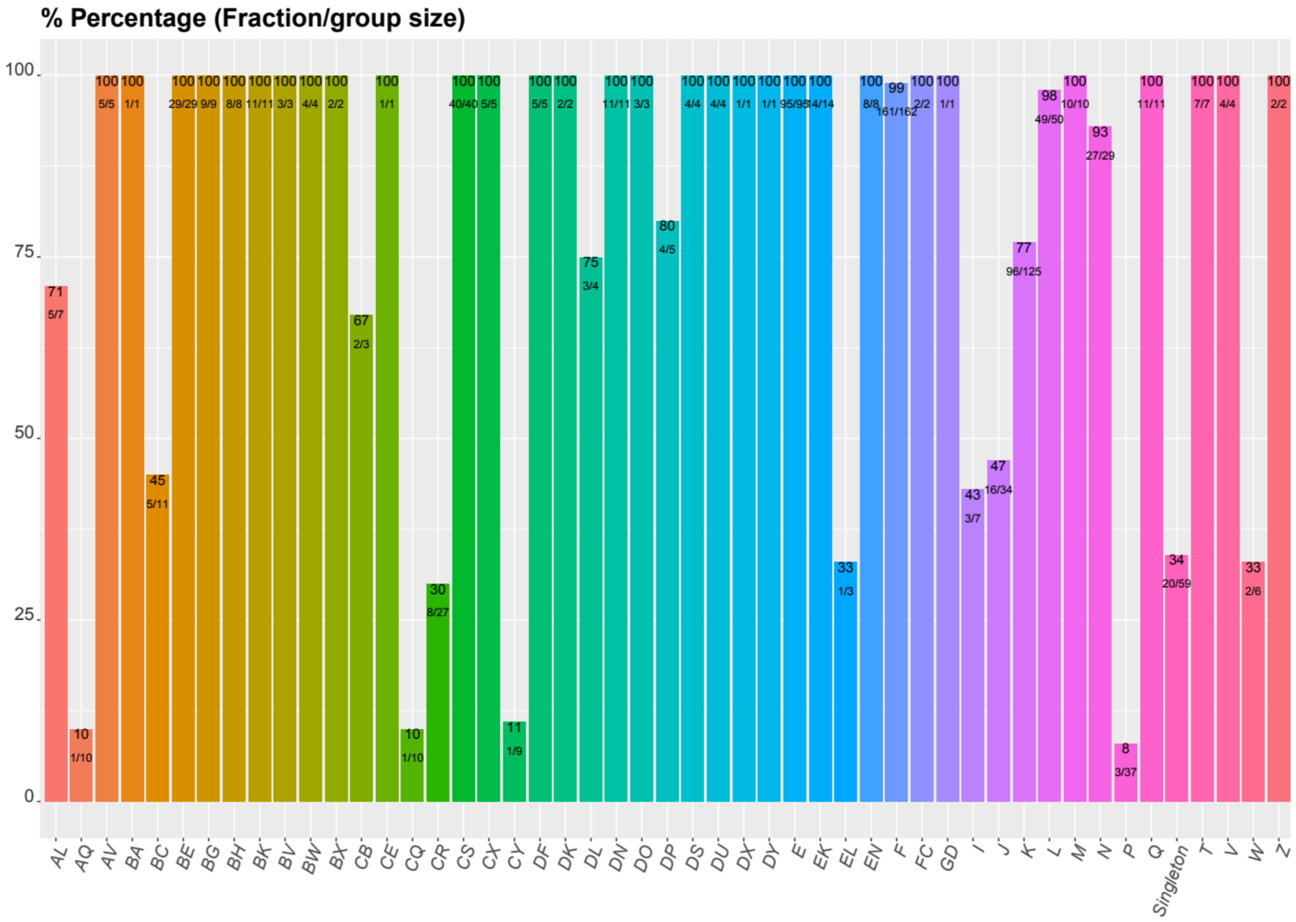

Fig. S4: Bar-plot representing the actual count and proportion of WhiB-encoding phages according to the known clusters.

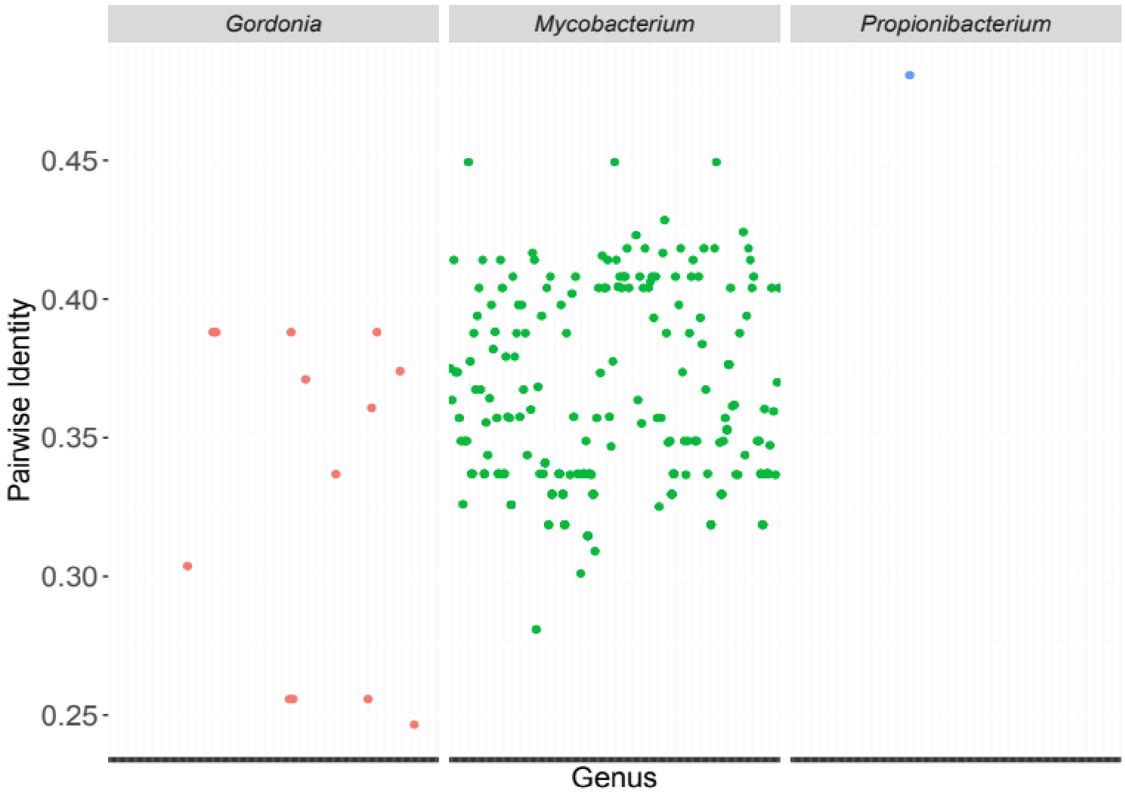

Fig.S5: Dot-plot represents the global protein pairwise sequence identity between phage-encoded *whiB* gene copies per genome according to their host genus.

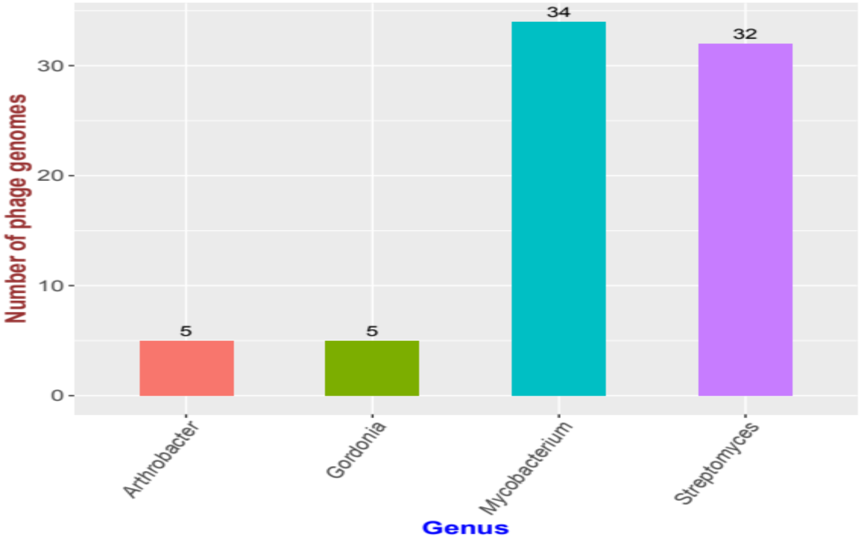

Fig. S6: Bar-plot displays the distribution of actinobacteriophage genomes that encode two copies of *lsr2* genes according to their host genus.

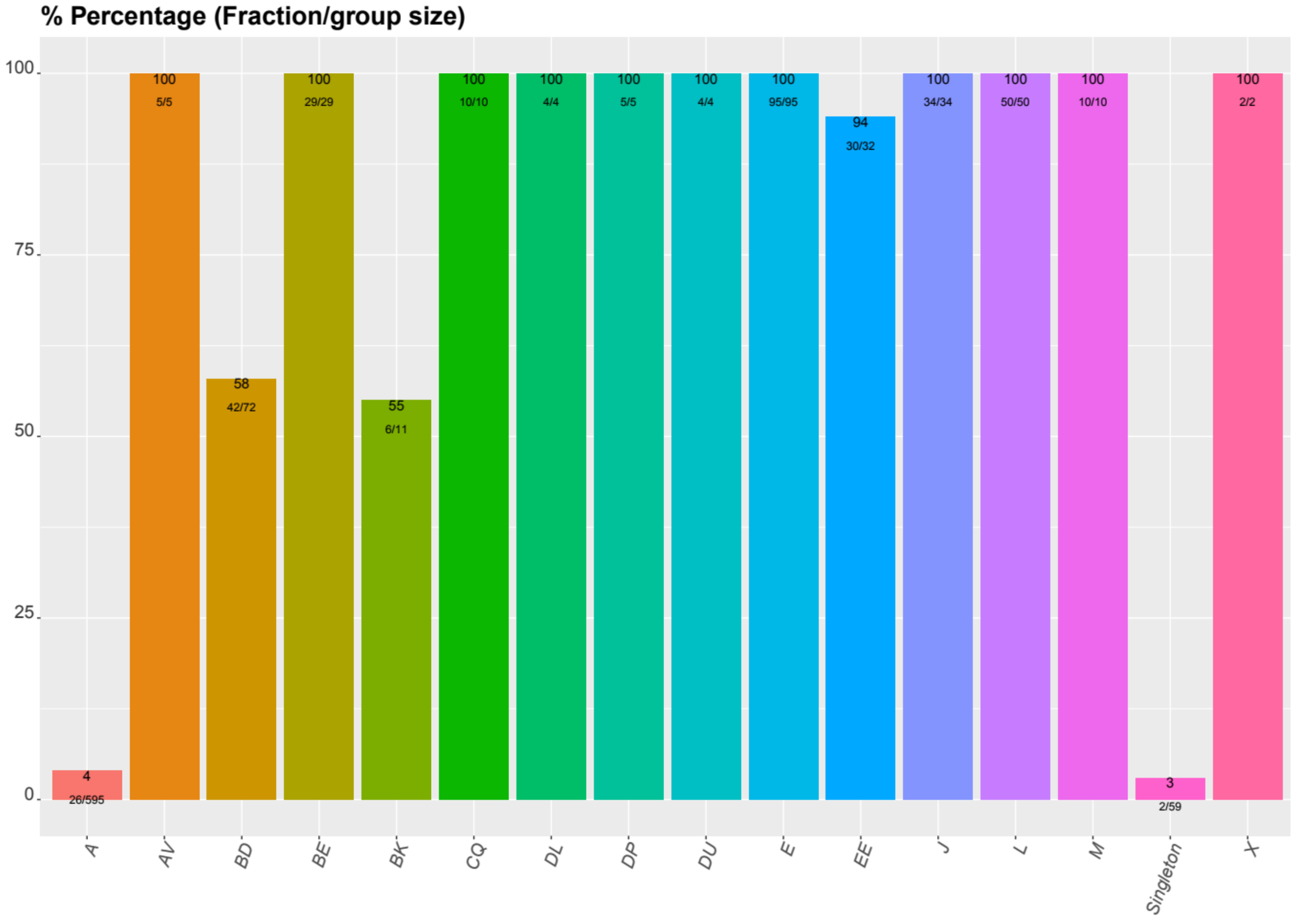

Fig. S7: Bar-plot representing the actual count and proportion of Lsr2-encoding phages according to the known clusters.

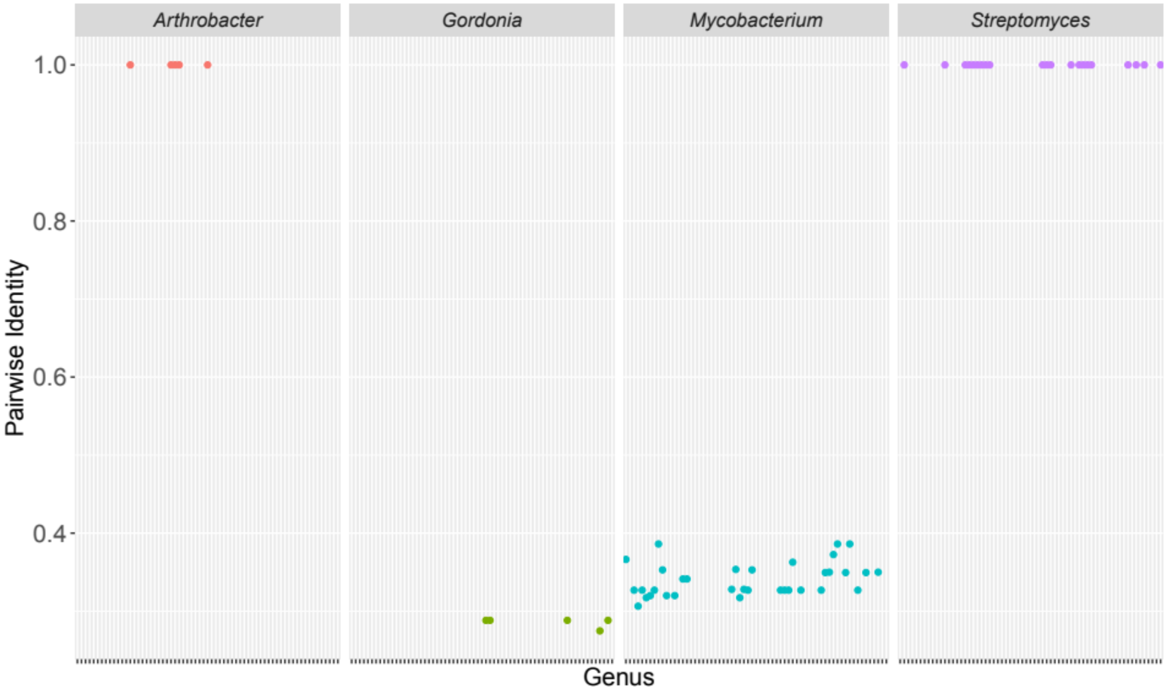

Fig.S8: Dot-plot represents the global protein pairwise sequence identity between phage-encoded *lsr2* gene copies per genome according to their host genus. Please note, in the case of *Athrobacter* and *Streptomyces*, the *lsr2* gene copies are found in the direct repeat regions of the phages, sharing 100% of sequence identity.

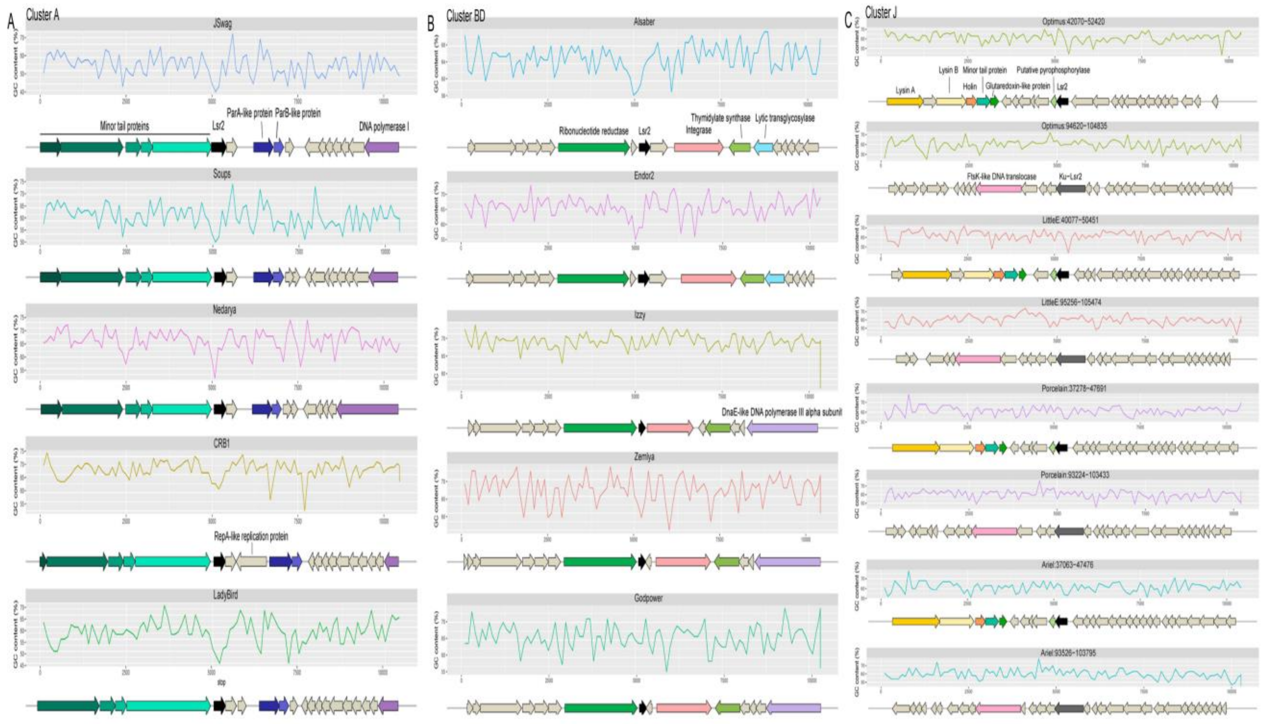

Fig. S9.A-C: Gene synteny plot showing the 5 kb regions flanking *lsr2* across randomly selected representative phage genomes of cluster A, BD and J, following the cluster assignment determined by phagesDB (2). GC content is displayed above the gene synteny plot for all genomes.

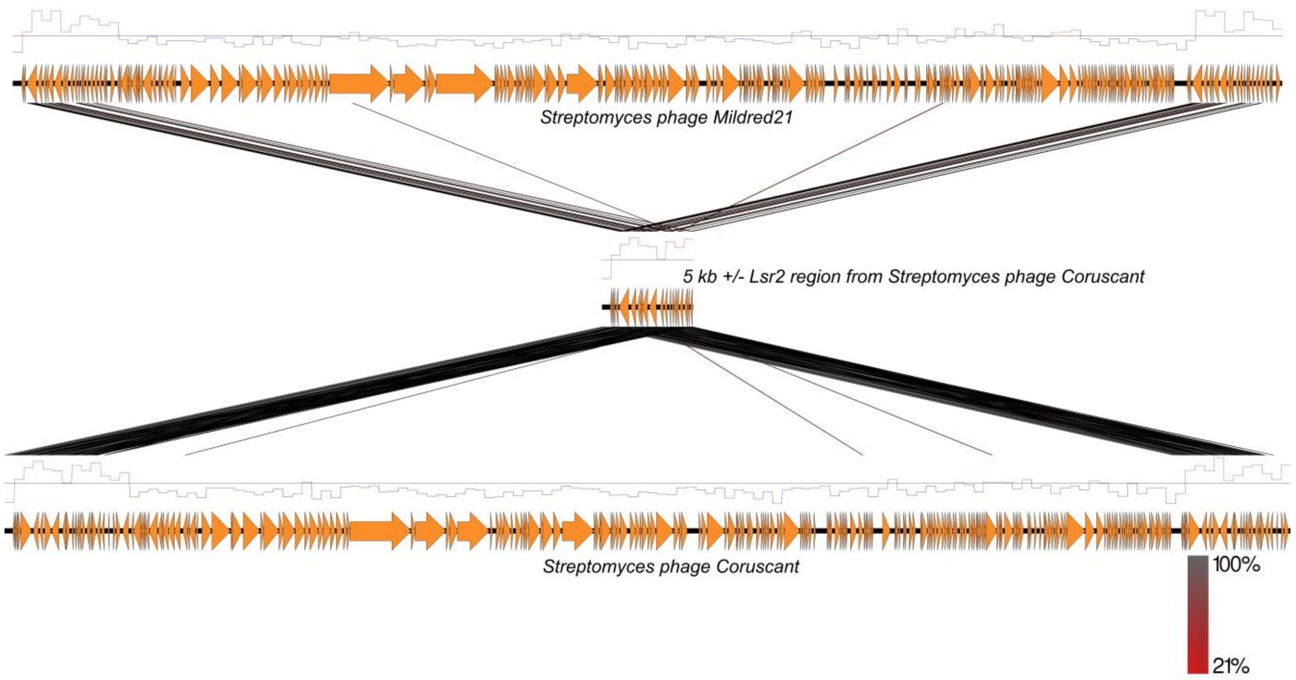

Fig. S10: Lsr2 homologs encoded by *Streptomyces* phages of the BE cluster are located in GC-rich, long direct terminal repeats. Genome comparison of two *Streptomyces* phages (Mildred21 & Coruscant) against the 10-kb region centered around Lsr2, visualized using Easyfig v 2.2.2 software (3). Gray shades show conserved regions with a high level of sequence identity, as calculated based on tBLASTx (4). Arrows indicate open reading frames (ORFs), and GC content is indicated on top of the genomes.

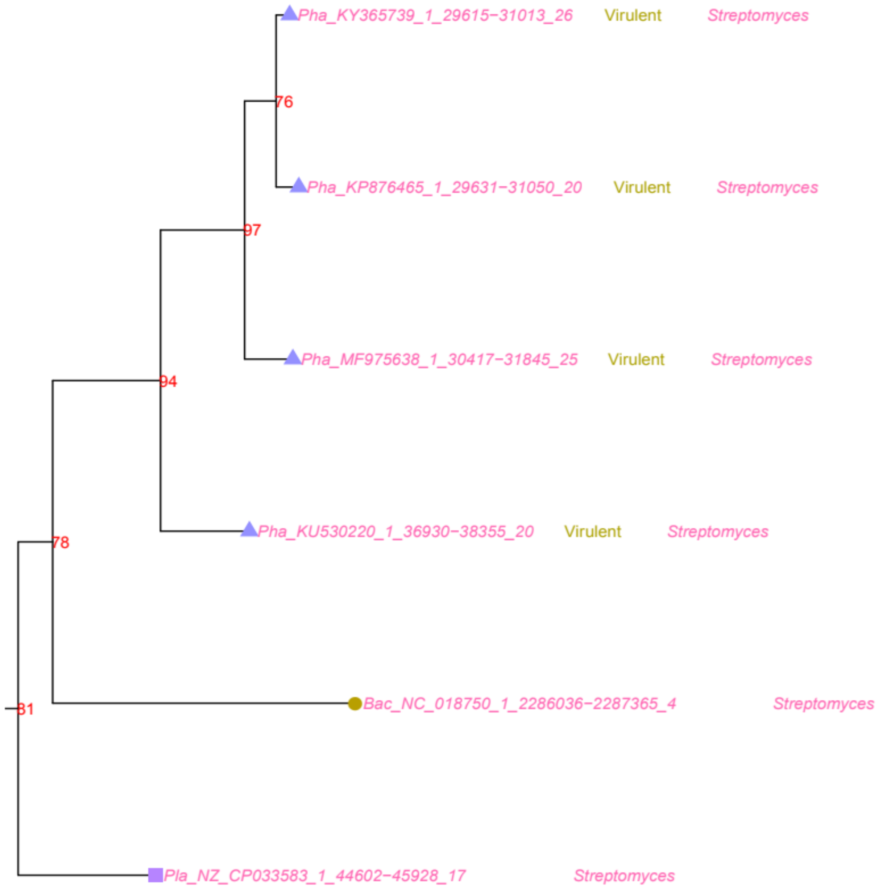

Fig. S11: WhiB phylogenetic subclade suggesting the acquisition of *whiB* by virulent *Streptomyces* phages from their host species. Tree tips shape indicate sequence source (bacteria: circle, phage: triangle, and plasmid: square). Sequences are color-coded according to the host genus. Phage lifestyle and genus information are shown on the right. Confidence values displayed on the nodes were estimated based on SH test using FastTree (5).

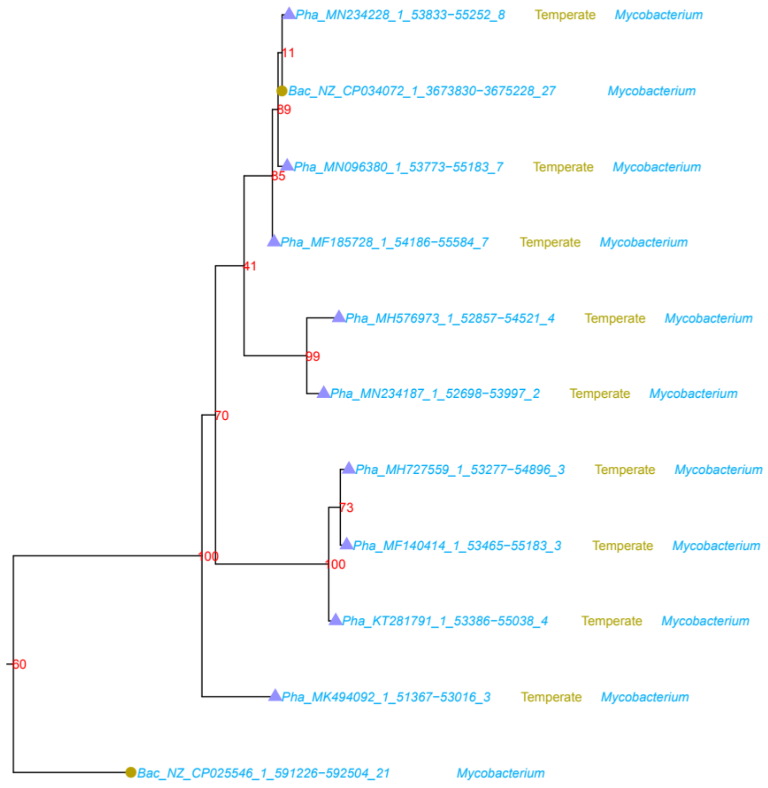

Fig. S12: WhiB phylogeny subclade suggesting the acquisition of *whiB* by temperate *Mycobacterium* phages from their host species. Tree tips shape indicate sequence source (bacteria: circle, and phage: triangle). Sequences are color-coded according to the host genus. Phage lifestyle and genus information are shown on the right. Confidence values displayed on the nodes were estimated based on SH test using FastTree (5).

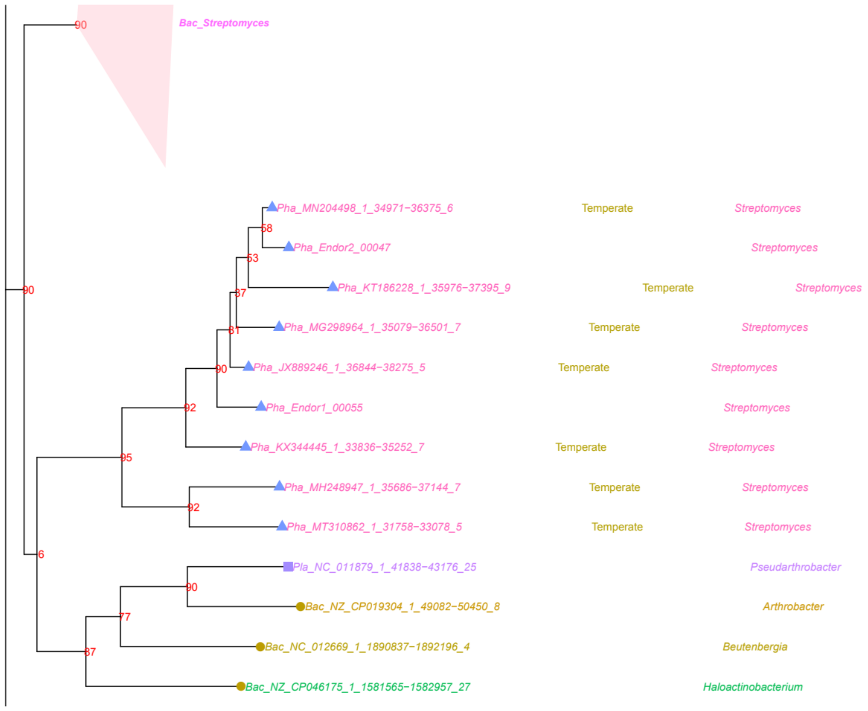

Fig. S13: Lsr2 phylogeny subclade suggesting the acquisition of *lsr2* by temperate *Streptomyces* phages from their hosts. Tree tips shape indicate sequence source (bacteria: circle, phage: triangle, and plasmid: square). Sequences are color-coded according to the phage lifestyle and host genus. Phage lifestyle and genus information are shown on the right. Confidence values displayed on the nodes were estimated based on SH test using FastTree (5).

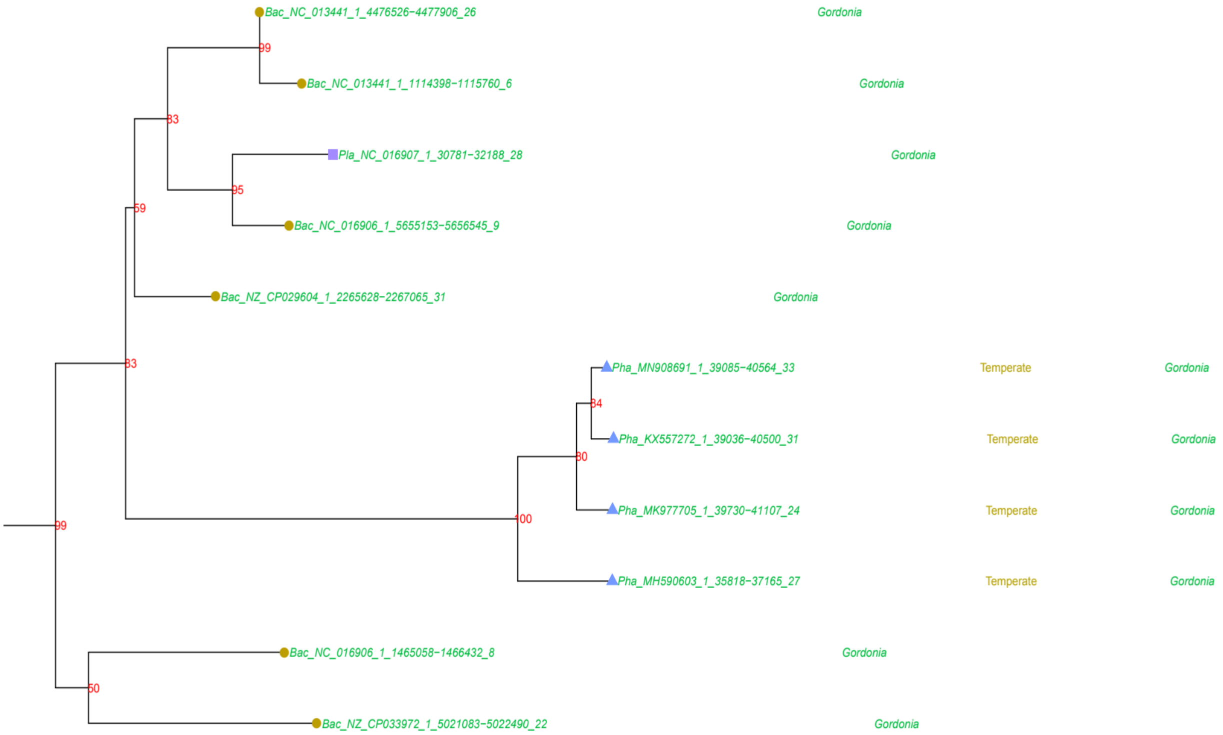

Fig. S14: Lsr2 phylogeny subclade suggesting the acquisition of *lsr2* by temperate *Gordonia* phages from their host species. Tree tips shape indicate sequence source (bacteria: circle, phage: triangle, and plasmid: square). Sequences are color-coded according to the host genus. Phage lifestyle and genus information are shown on the right. Confidence values displayed on the nodes were estimated based on SH test using FastTree (5).

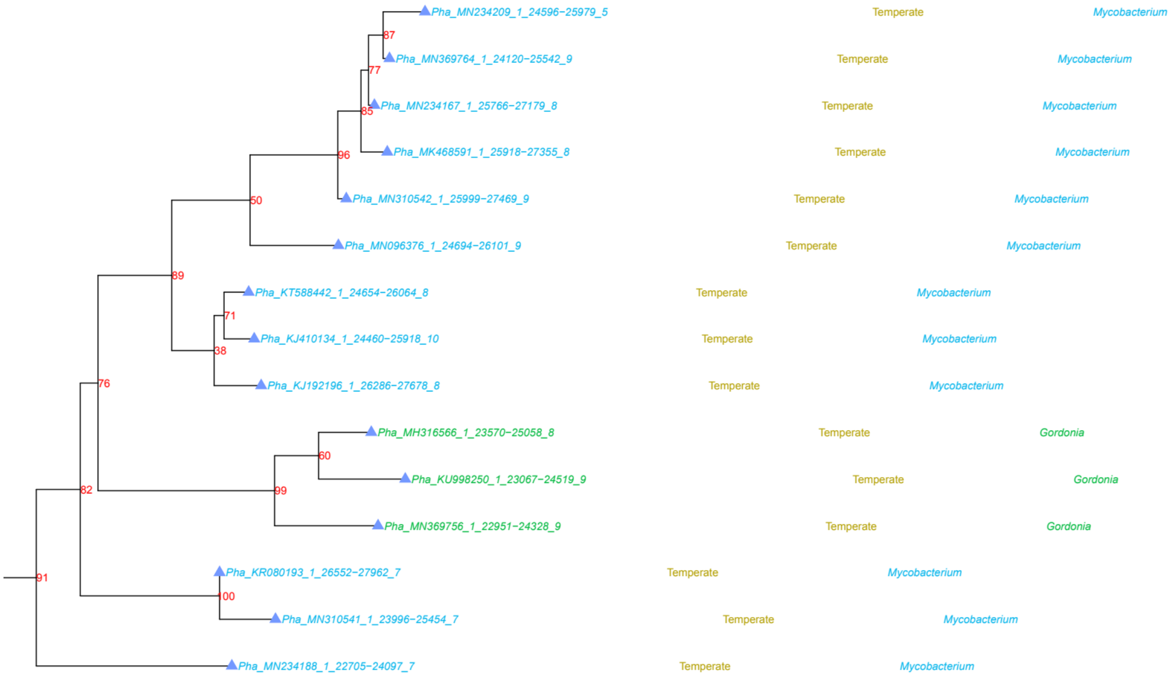

Fig.15: Lsr2 phylogeny subclade suggesting a transfer of *lsr2* from temperate *Gordonia* phages to *Mycobacterium* phages. Tree tips shape indicate sequence source (bacteria: circle, phage: triangle, and plasmid: square). Sequences are color-coded according to the host genus. Phage lifestyle and genus information are shown on the right. Confidence values displayed on the nodes were estimated based on SH test using FastTree (5).

Legends of supplementary tables

Table S1: The 2951 actinobacteriophage genomes used in the current study with corresponding information such as genome size, lifestyle, GC content, assigned clusters, etc (downloaded from phagesdb.org).

Table S2; Sheet1: Identified thirteen regulatory protein domains distribution across actinobacteriophage genomes. Merged overlapping domains are highlighted in bold.

Table S2; Sheet2: List of identified ninety-four manually curated regulatory protein domains; distribution across actinobacteriophage genomes.

Table S3: List of genes and their quantitative distribution within 5 kb flanking *whiB* genomic loci regions identified within the actinobacteriophage genomes according to assigned clusters.

Table S4: Summary of the gene domains within 5 kb flanking *whiB* genomic loci regions identified within the actinobacteriophage genomes according to assigned clusters.

Table S5: List of gene domains and their quantitative distribution within 5 kb flanking *lsr2* genomic loci regions identified within the actinobacteriophage genomes according to assigned clusters.

Table S6: Summary of the gene domains within 5 kb flanking *lsr2* genomic loci regions identified within the actinobacteriophage genomes according to assigned clusters.

REFERENCES

1. Sims GE, Jun S-R, Wu GA, Kim S-H. 2009. Alignment-free genome comparison with feature frequency profiles (FFP) and optimal resolutions. Proc Natl Acad Sci 106:2677–2682.
2. Russell DA, Hatfull GF. 2017. PhagesDB: the actinobacteriophage database. Bioinformatics 33:784–786.
3. Sullivan MJ, Petty NK, Beatson SA. 2011. Easyfig: a genome comparison visualizer. Bioinformatics 27:1009–1010.
4. Altschul SF, Gish W, Miller W, Myers EW, Lipman DJ. 1990. Basic local alignment search tool. J Mol Biol 215:403–410.
5. Price MN, Dehal PS, Arkin AP. 2010. FastTree 2 – Approximately Maximum-Likelihood Trees for Large Alignments. PLoS One 5:e9490.
